# Supplementary material for: RIF1-ASF1-mediated high-order chromatin structure safeguards genome integrity
Source: Nat Commun. 2022 Feb 17;13:957. doi: 10.1038/s41467-022-28588-y (PMC8854732; doi:10.1038/s41467-022-28588-y)
Supplement: Supplementary file 1 — Supplementary information [file 41467_2022_28588_MOESM1_ESM.pdf]

Supplementary information

RIF1-ASF1-mediated high-order chromatin structure safeguards genome integrity

Sumin Feng[1,3], Sai Ma[1,3], Kejiao Li[1,3], Shengxian Gao[1], Shaokai Ning[1], Jinfeng Shang[1], Ruiyuan Guo[1], Yingying Chen[1], Britny Blumenfeld[2], Itamar Simon[2], Qing Li[1], Rong Guo[1], and Dongyi Xu[1*].

[1]State Key Laboratory of Protein and Plant Gene Research, School of Life Sciences, Peking University, Beijing, China 100871.

[2]Department of Microbiology and Molecular Genetics, Institute of Medical Research Israel-Canada, Faculty of Medicine, The Hebrew University, Jerusalem 91120, Israel.

[3]These authors contributed equally to this work.

* Corresponding author: xudongyi@pku.edu.cn.

This PDF file contains:

Supplementary Figure 1-14

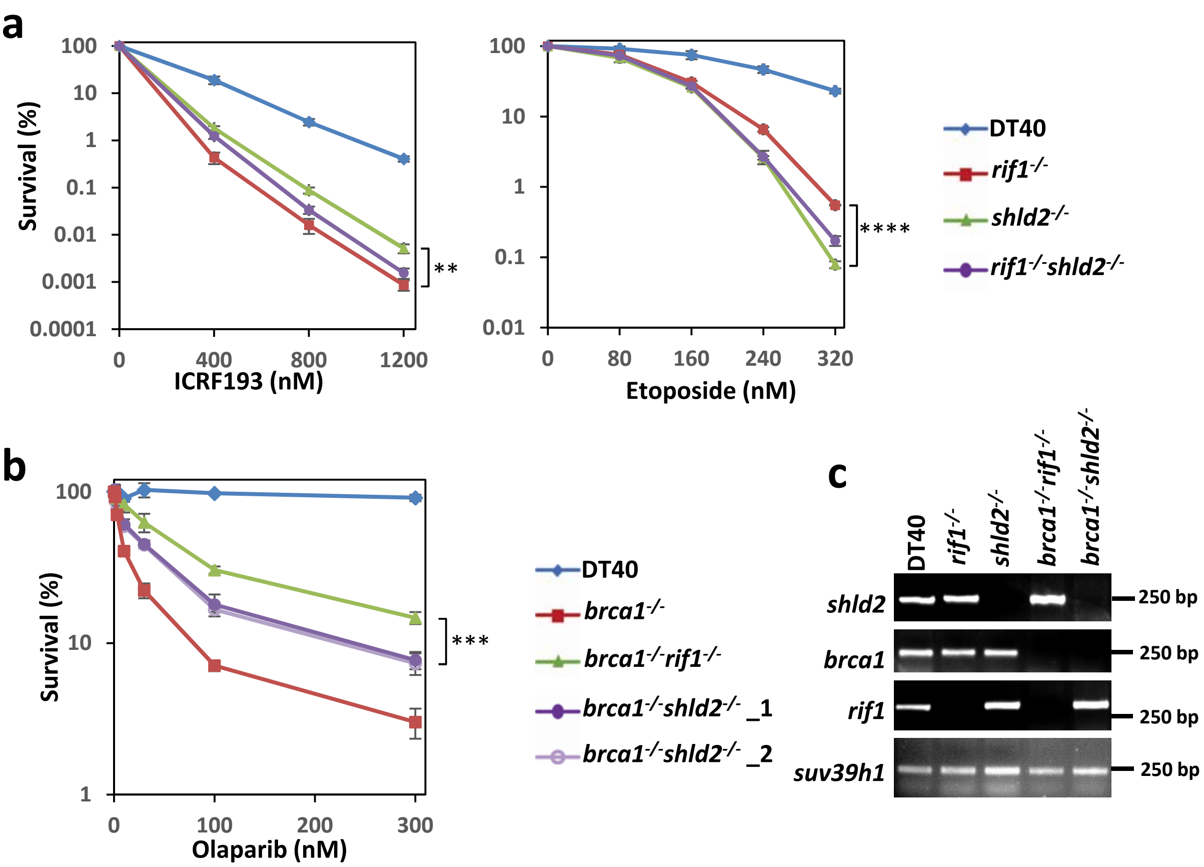

Supplementary Figure 1: SHLD2 is not as effective as RIF1 to promote NHEJ and suppress resection in BRCA1-deficient cells.

a, Etoposide- or ICRF193-sensitivity of various groups of DT40 cells using colony formation assay. The mean and s.d. from three independent experiments are shown. **b,** The graphic shows the PARPi (olaparib) -sensitivity of BRCA1-deficient DT40 cells when RIF1 or SHLD2 was knockout. Two *brca1*$^{-/-}$*shld2*$^{-/-}$ clones were used. The mean and s.d. from three independent experiments are shown. **, p<0.01; ***, p<0.001; ****, p<0.0001. Statistical analysis was performed using two-way *t*-test (a) or one-way ANOVA (b). **c,** Cell lines validation by genomic PCR (*shld2* primers: GGAGACAGAAAGATGGAGTAAG and TCTACTTGAGAGCACAGTATTC; *brca1* primers : GGAAGCTATCAGTGCGTTTGAGCTTGATAC and GGAGCTCAGCAGTAGCAACTTCTGTGGATG; *rif1* primers : GGAACTTCAGAAATTGTTTTCTAC and ATCTATTAGACTCTTCCATGCAATG; *suv39h1* primers: TTCGTCTACATCAACGAGTACAAAGT and AAGATGCAGAGGTCGTAGCGGAT). Uncropped images of the gels and statistical source data including the precise *P* values are provided in Source data.

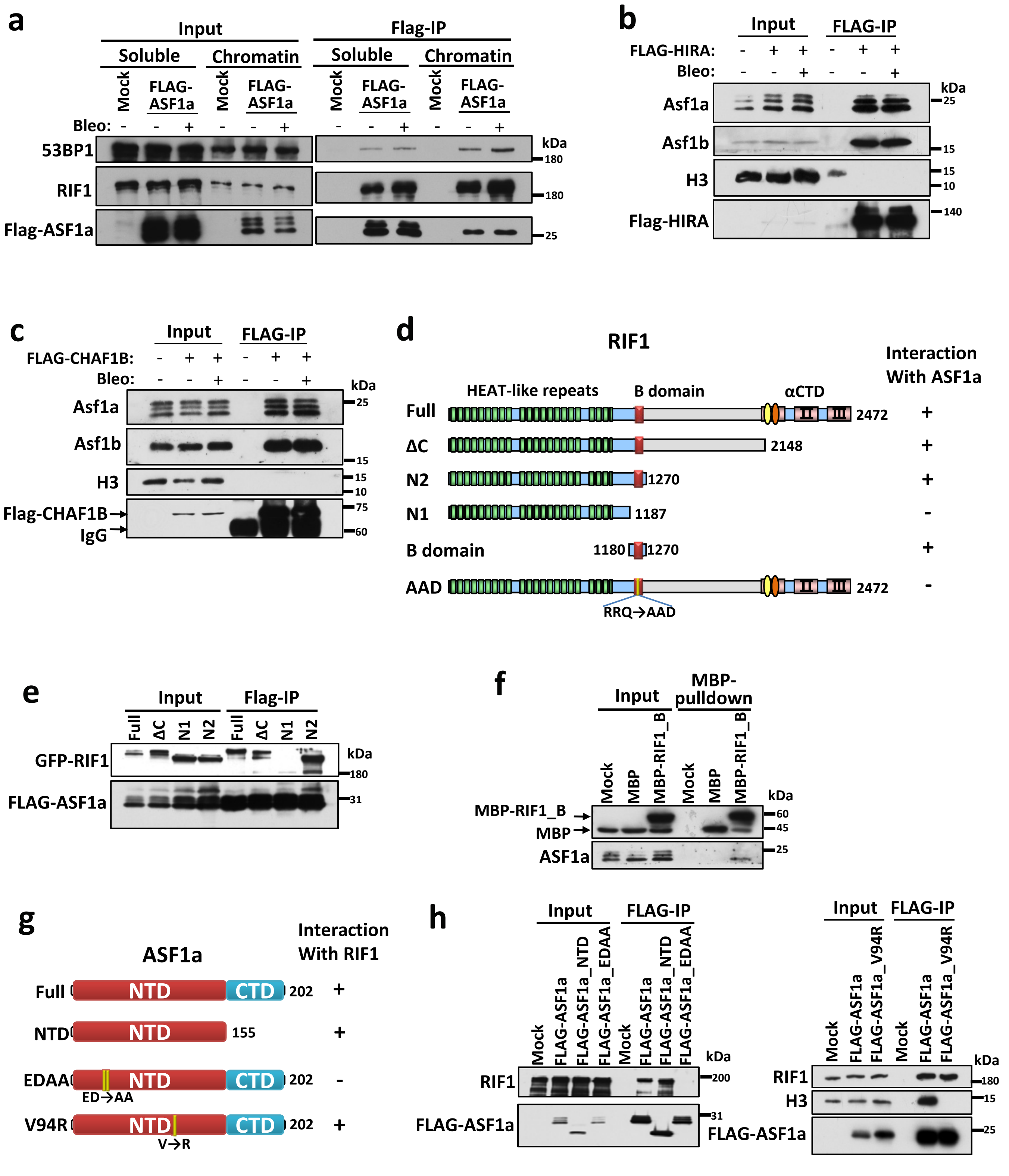

Supplementary Figure 2: RIF1 interacts with ASF1a in a manner similar to HIRA and CAF-1.
a, Immunoblot showing IP of FLAG-tagged ASF1a using soluble or chromatin fractions. Cells were treated with/without bleomycin (20 μg/mL) for 3 h before harvest. **b, c,** Immunoblot showing IP of FLAG-tagged HIRA (b) and CAF-1 p60 (CHAF1B) (c). **d,** Schematic representations of different RIF1 mutants (left) and their ability to coimmunoprecipitate with ASF1a (right). **e, f,** Immunoprecipitation (e) and MBP-pulldown (f) to assess whether the various deletion mutants of RIF1 co-purified with ASF1a. **g,** Schematic representations of different ASF1a mutants (left) and their ability to coimmunoprecipitate with RIF1 (right). EDAA, E36A/D37A. **h,** Immunoprecipitation to assess whether the various mutants of ASF1a co-purified with RIF1.

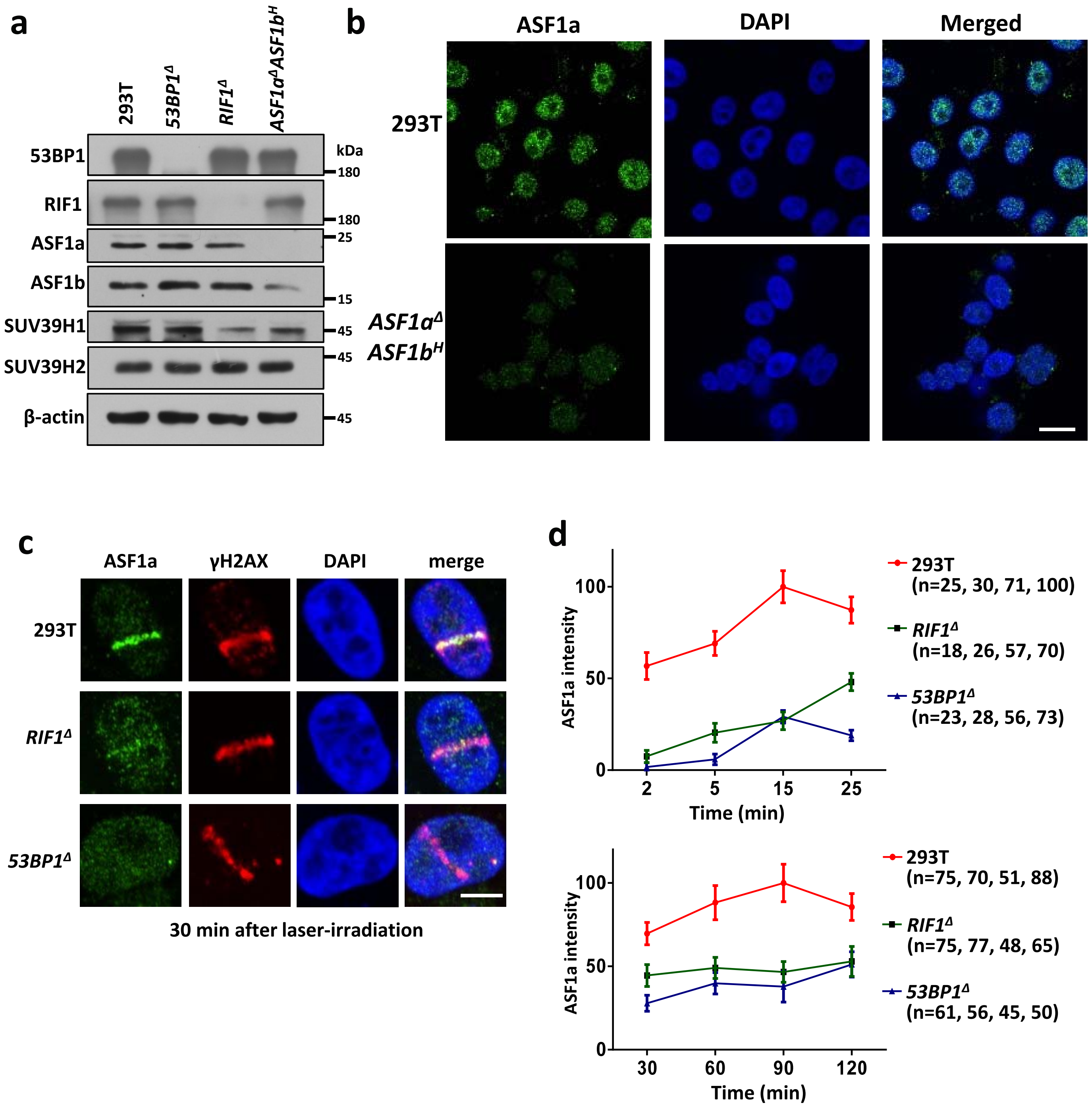

Supplementary Figure 3: ASF1a is recruited to DNA damage sites by 53BP1 and RIF1.
a, Immunoblots showing protein levels in knockout HEK293T cells. **b**, Validation of anti-ASF1a antibodies in immunostaining. Scale bar, 10 µm. **c, d,** Immunostaining (c) of ASF1a in wild-type, *53BP1*$^{\Delta}$ or *RIF1*$^{\Delta}$ HEK293T cells and quantification (d) after laser-induced DNA damage. Mean ± SEM is shown for every time point. Scale bar, 5 µm.

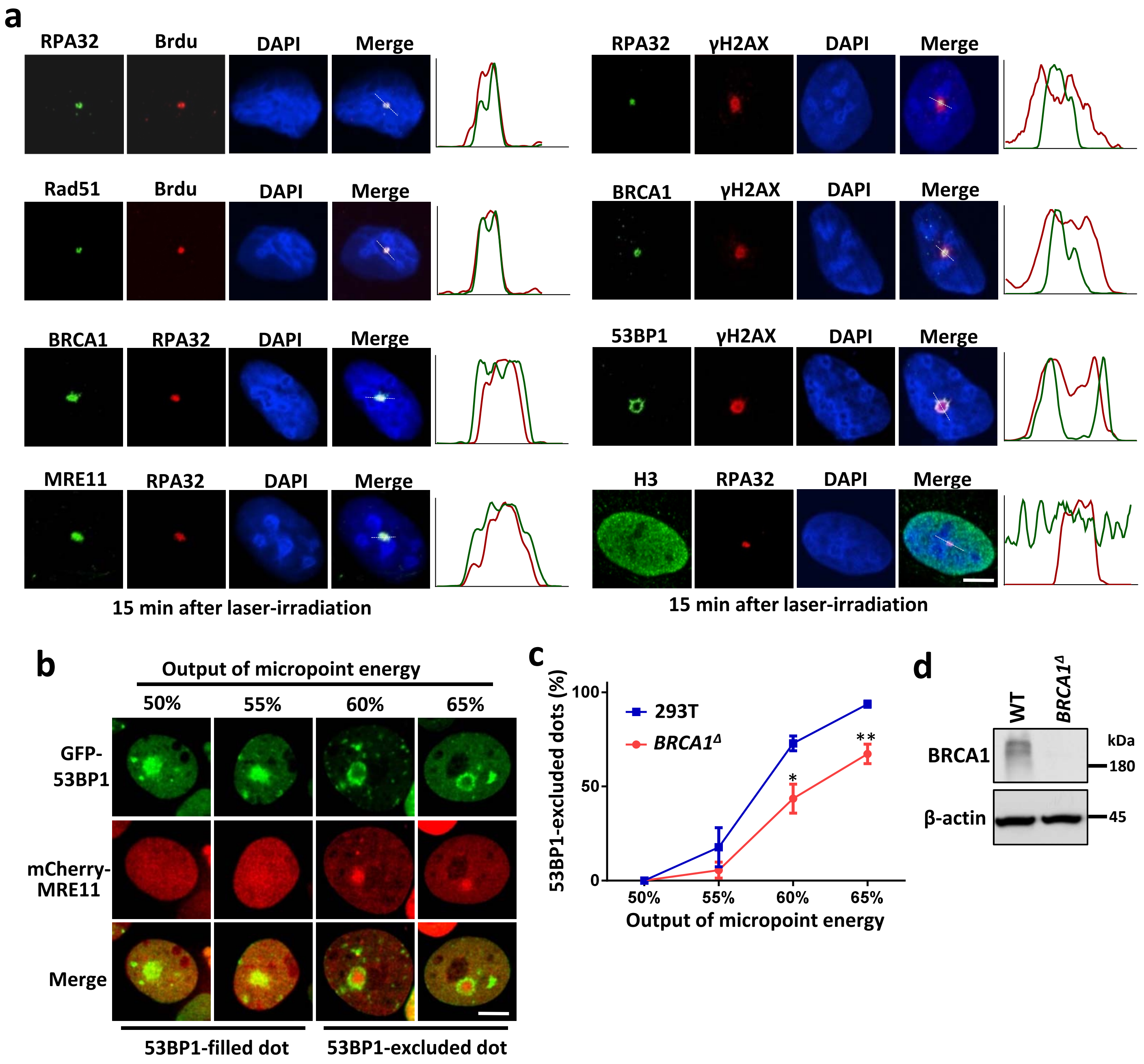

Supplementary Figure 4: Exclusion of 53BP1 from the core irradiated region is dose-dependent and promoted by BRCA1.

a, Immunofluorescence showing the distribution of multiple proteins in the HIRDC assay. For ssDNA staining, cells were incubated with BrdU for 24 hours before micro-irradiation. The right panels for every image show the intensity distribution of the red and green signals on the white dashed line indicated in the image. **b,** GFP-53BP1 in the HIRDC assay 15 min after various doses of laser energy in HEK293T cells. **c,** Quantification of the distribution pattern of GFP-53BP1 in the HIRDC assay in wild-type or *BRCA1*$^{\Delta}$ HEK293T cells. Two patterns of GFP-53BP1 distribution were defined as in (b). Scale bar, 5 μm. The mean and s.d. from three independent experiments are shown. *, $p<0.05$; **, $p<0.01$; Statistical analysis was performed using the two-tailed *t*-test. **d,** Immunoblots showing protein level of BRCA1 in knockout cells. Uncropped images of the gels and statistical source data including the precise *P* values are provided in Source data

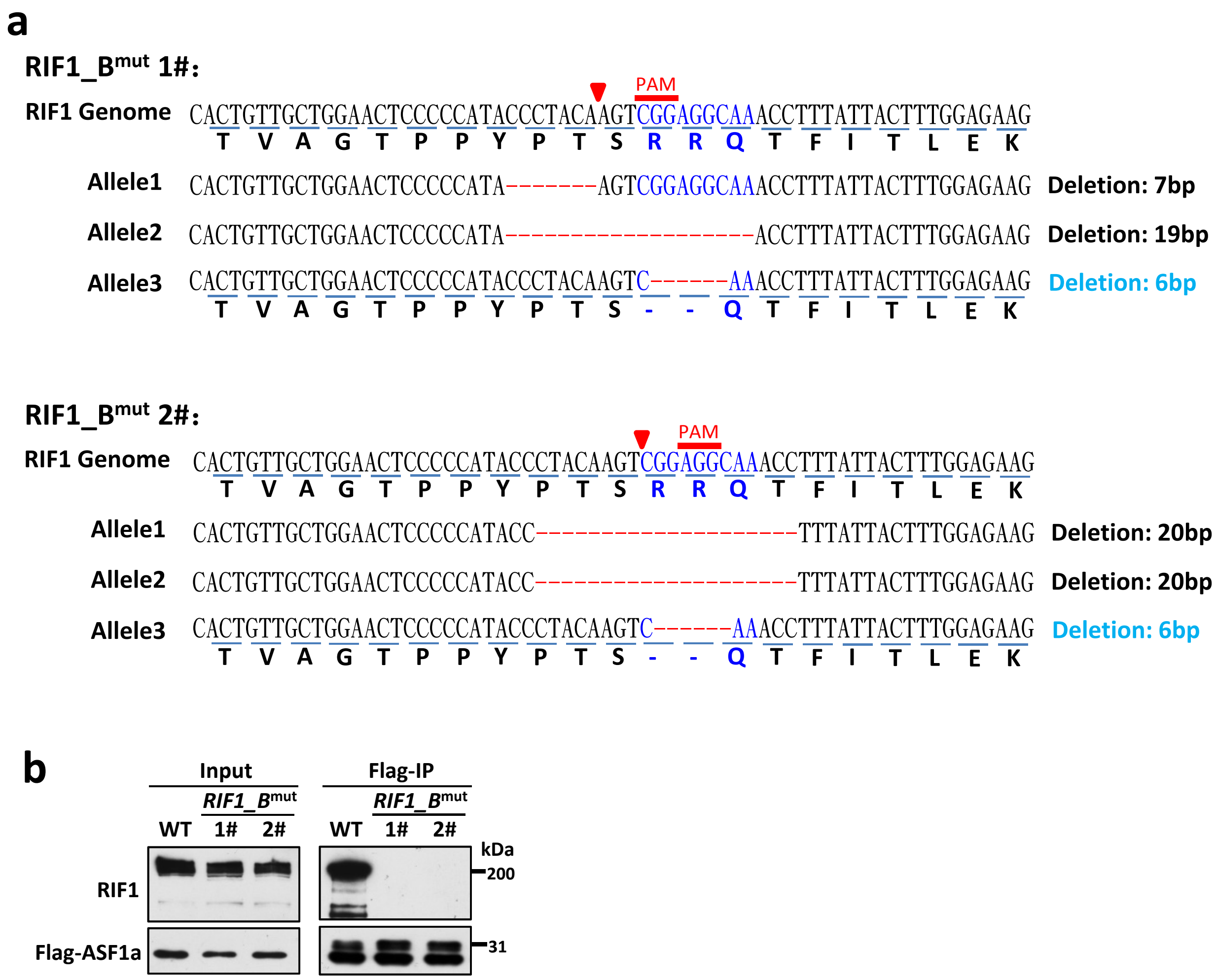

Supplementary Figure 5: Generation of *RIF1_B^{mut}* HEK293T cells.

a, Schematic representation of the generation of *RIF1_B^{mut}* HEK293T cells using CRISPR. PAM sequences are indicated by red lines. The red arrow indicates a putative cleavage site. Red dashes indicate deleted bases. **b**, Immunoblot showing IP of FLAG-tagged ASF1a in wild type or *RIF1_B^{mut}* HEK293T cells. The wild type or *RIF1_B^{mut}* HEK293T cells were transfected with plasmid expressing FLAG-tagged ASF1a before IP. RIF1_B^{mut}, which lacks conserved 1217R1218R in the B domain, fully lost its interaction with ASF1a.

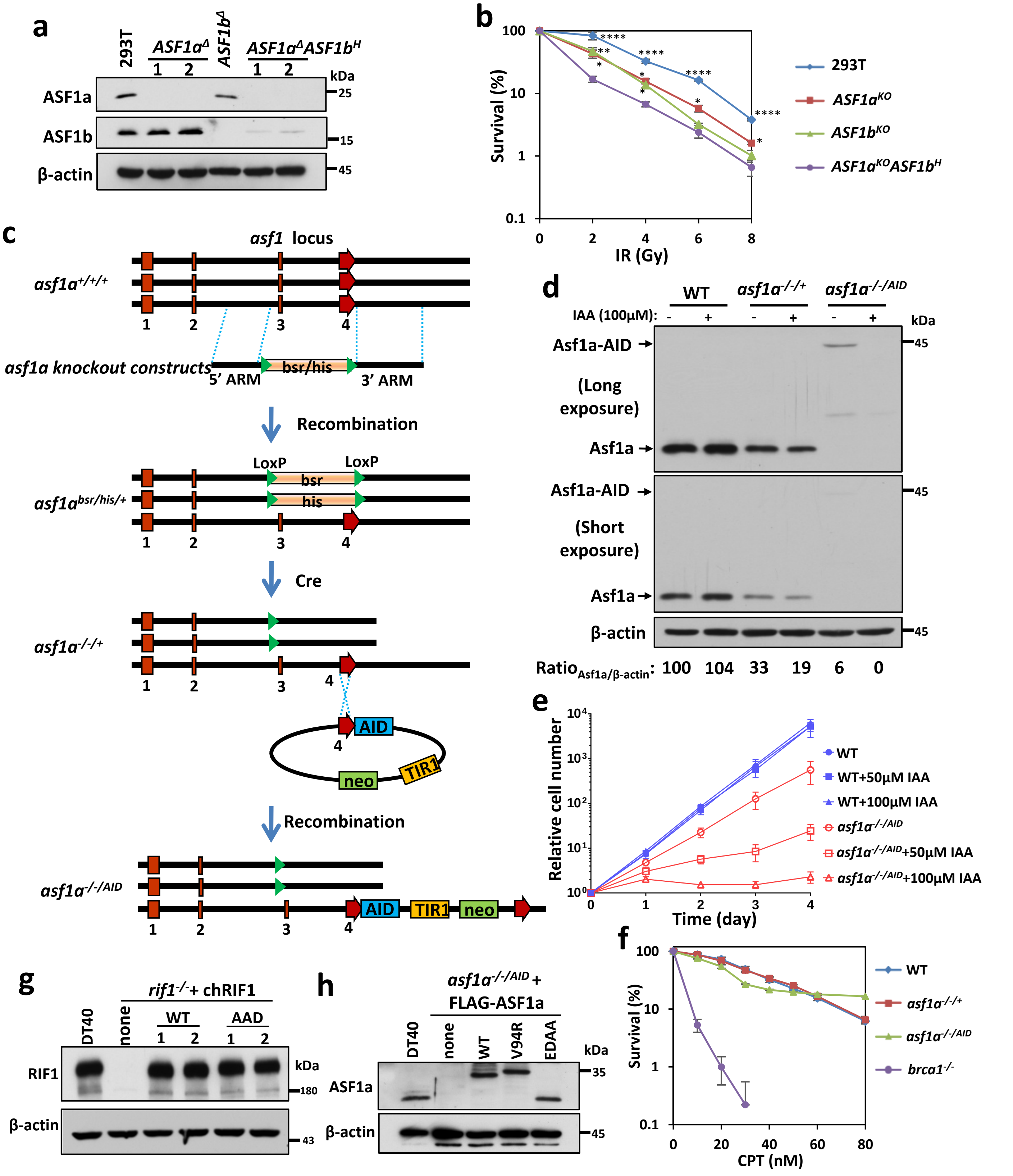

Supplementary Figure 6: Generation of ASF1a knockout HEK293T and DT40 cells.

a, Immunoblots showing the expression levels of ASF1a and ASF1b in knockout cells. **b,** IR sensitivity assay for *ASF1a*$^{\Delta}$, *ASF1b*$^{\Delta}$ and *ASF1a*$^{\Delta}$*ASF1b*^{H} HEK293T cells. The mean and s.d. of the results from three independent experiments are shown. Statistical analysis between *ASF1a*^{KO}*ASF1b*^{H} with other cells was performed using the two-way ANOVA. *, p<0.05; **, p<0.01; ****, p<0.0001. **c,** Schematic representation of the chicken wild-type and targeted genomic DNA in the *asf1a* gene. The regions containing exons (marked by red) of these genes were replaced by histidinol or blasticidin resistance genes. The two regions between two pairs of dotted lines were used as the arms of the knock-out constructs. The third allele was fused with AID at the C-terminal. **d,** Immunoblots showing the ASF1a level in the knockout cells with or without IAA. **e,** Growth curves of *asf1a*$^{-/-/AID}$ cells. The mean and s.d. from three independent experiments are shown. **f,** CPT-sensitivity assay of *asf1a*$^{-/-/+}$ and *asf1a*$^{-/-/AID}$ cells. The mean and s.d. of the results from three independent experiments are shown. **g, h,** Immunoblots showing RIF1 (g) and ASF1a (h) levels in their complemented cells.

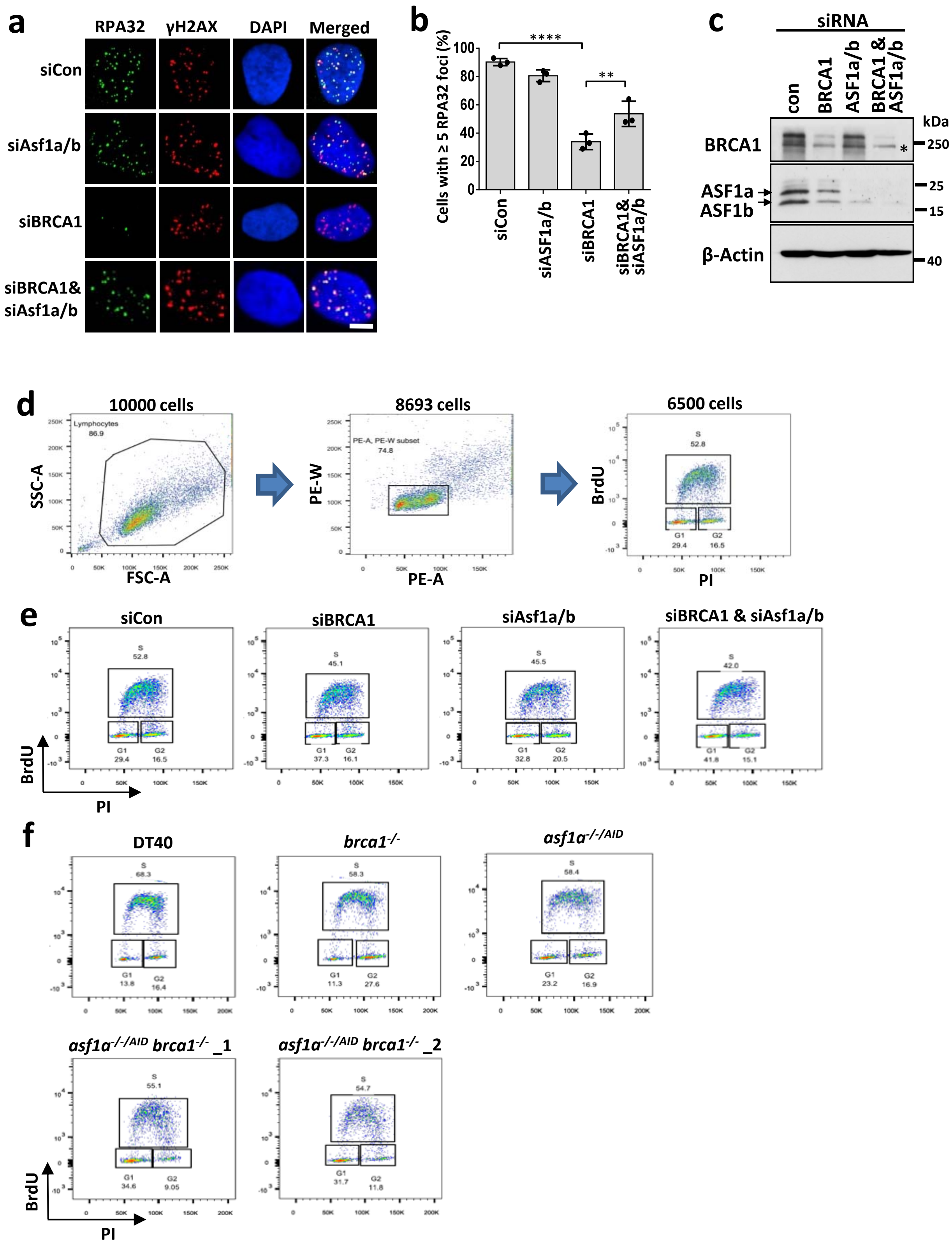

Supplementary Figure 7: ASF1 suppresses broken end resection in BRCA1-deficient cells.

a, b, Immunofluorescence (a) and quantification (b) of RPA32 foci. BRCA1- or/and ASF1a/b-depleted U2OS cells were treated with 25 Gy X-ray radiation 1 h before staining. The mean and s.d. from three independent experiments are shown. Scale bar, 5 μm. **, $p<0.01$; ****, $p<0.0001$. Statistical analysis was performed using the One-way ANOVA. **c,** Immunoblots showing the knockdown efficiency of BRCA1 and ASF1a/b in U2OS cells. Crossreactive polypeptides are indicated by an asterisk. **d,** A figure exemplifying the gating strategy. **e, f,** Cell cycle distribution of BRCA1- and ASF1-deficient cells. Acute depletion of ASF1 and/or BRCA1 did not change the cell cycle in U2OS cells (e). Depletion of ASF1a in the *brca1*$^{-/-}$ DT40 cells cause an increased population of G1 phase (f). Uncropped images of the gels and statistical source data including the precise *P* values are provided in Source data.

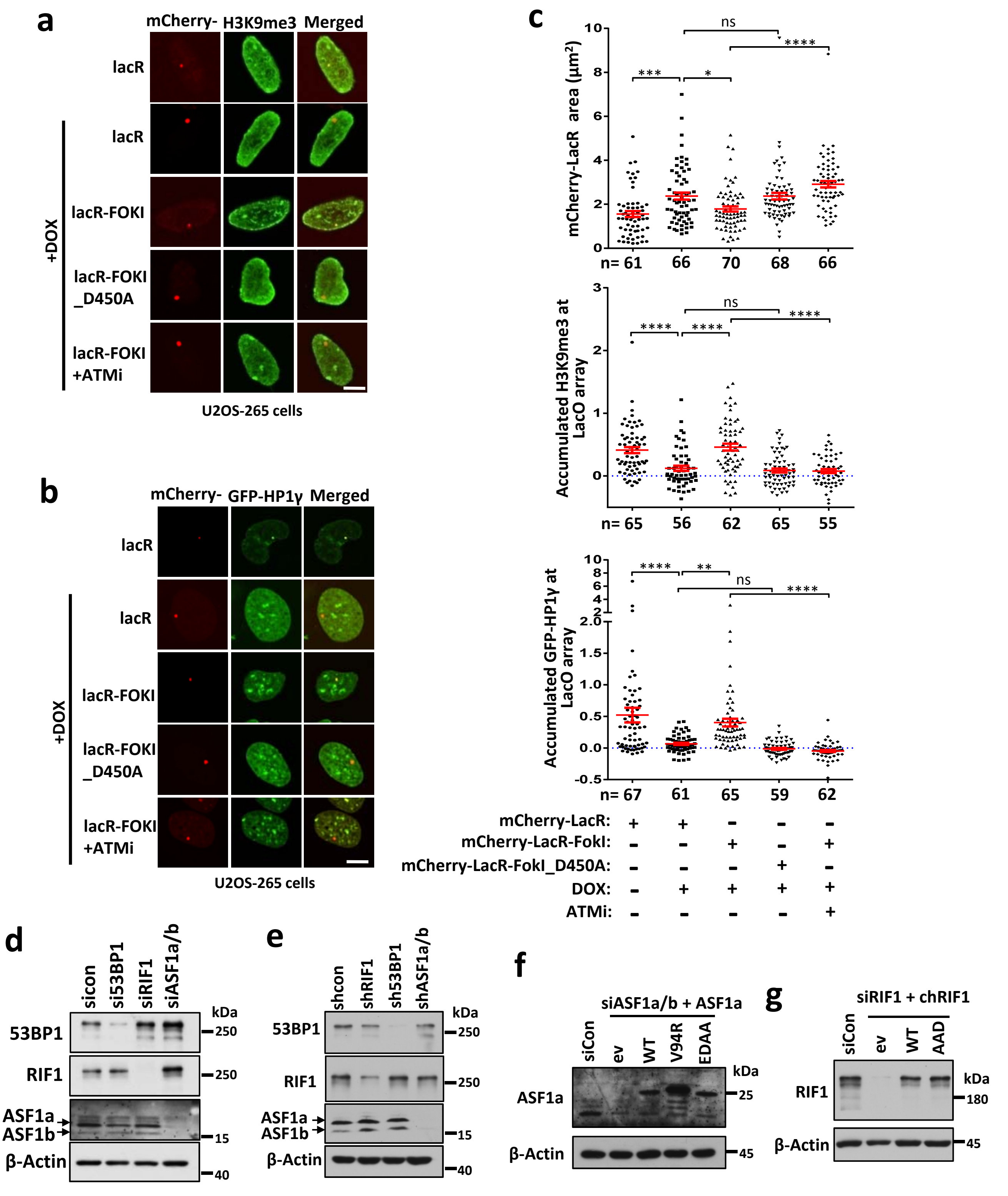

Supplementary Figure 8: DSBs induce chromatin condensation.

a-c, H3K9me3 (a) and GFP-HP1γ (b) signals in the array and their quantification (c) in U2OS-265 cells. Cells were transfected with mCherry-LacR, mCherry-LacR-fused wild-type or D450A mutant FokI. DOX and ATMi were added 5 hr and 3 hr before imaging, respectively. The mCherry-LacR area was also quantified in (c). Data are presented as mean values +/- SEM. The numbers of cells pooled from two independent experiments are indicated. **d, e,** Immunoblots showing the knockdown efficiency of 53BP1, RIF1 and ASF1a/b in U2OS-265 cells using siRNA (d) or shRNA (e). **f, g,** Immunoblots showing protein levels of ASF1a (f) and RIF1 (g) after complementation. Scale bar, 5 μm. ns, $p>0.05$; *, $p<0.05$; **, $p<0.01$; ***, $p<0.001$; ****, $p<0.0001$. Statistical analysis was performed using one-way ANOVA. Uncropped images of the gels and statistical source data including the precise *P* values are provided in Source data.

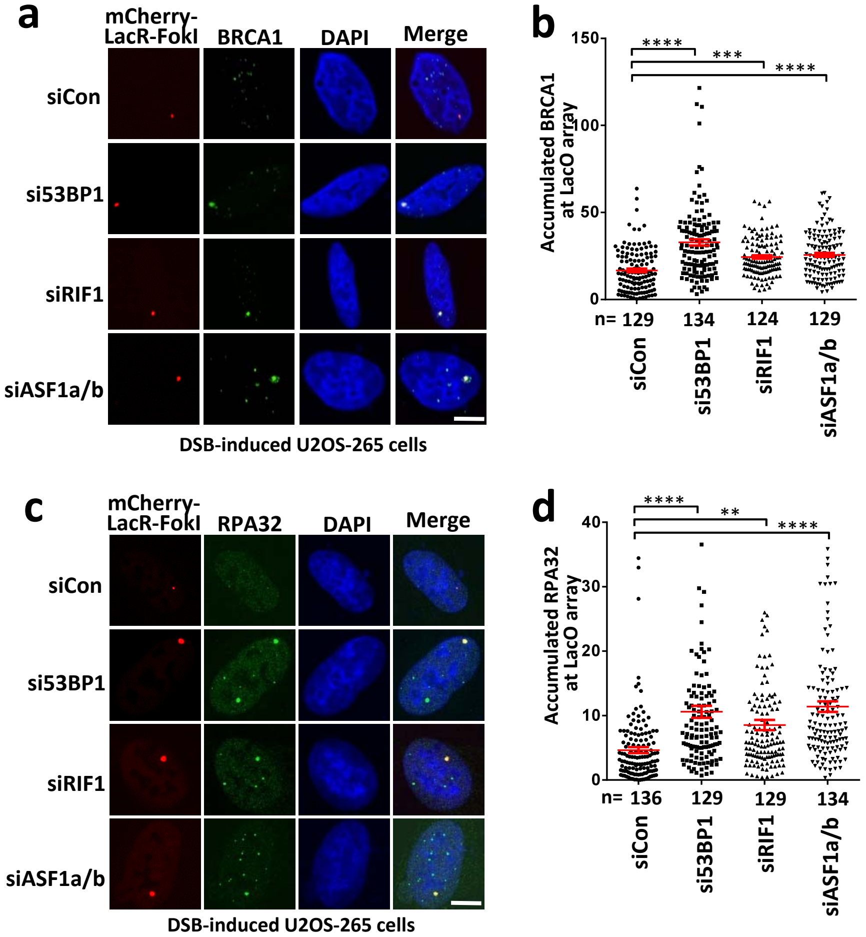

Supplementary Figure 9: 53BP1, RIF1 and ASF1a/b suppress the recruitment of BRCA1 and RPA to DSB sites.

a-d, Immunofluorescence of BRCA1 (a) and RPA32 (c) in the array of DSB-induced USOS-265 cells after depleting 53BP1, RIF1 or ASF1a/b and their quantification (b, d). Cells were synchronized in G1 phase before staining as described previously (*Nucleic Acids Research* 2013, Gamper, A.M. *et al*). Data are presented as mean values +/- SEM. The numbers of cells pooled from three independent experiments are indicated. Scale bar, 5 µm. **, p<0.01; ***, p<0.001; ****, p<0.0001. Statistical analysis was performed using the one-way ANOVA. Statistical source data, including the precise *P* values, are provided in the source data.

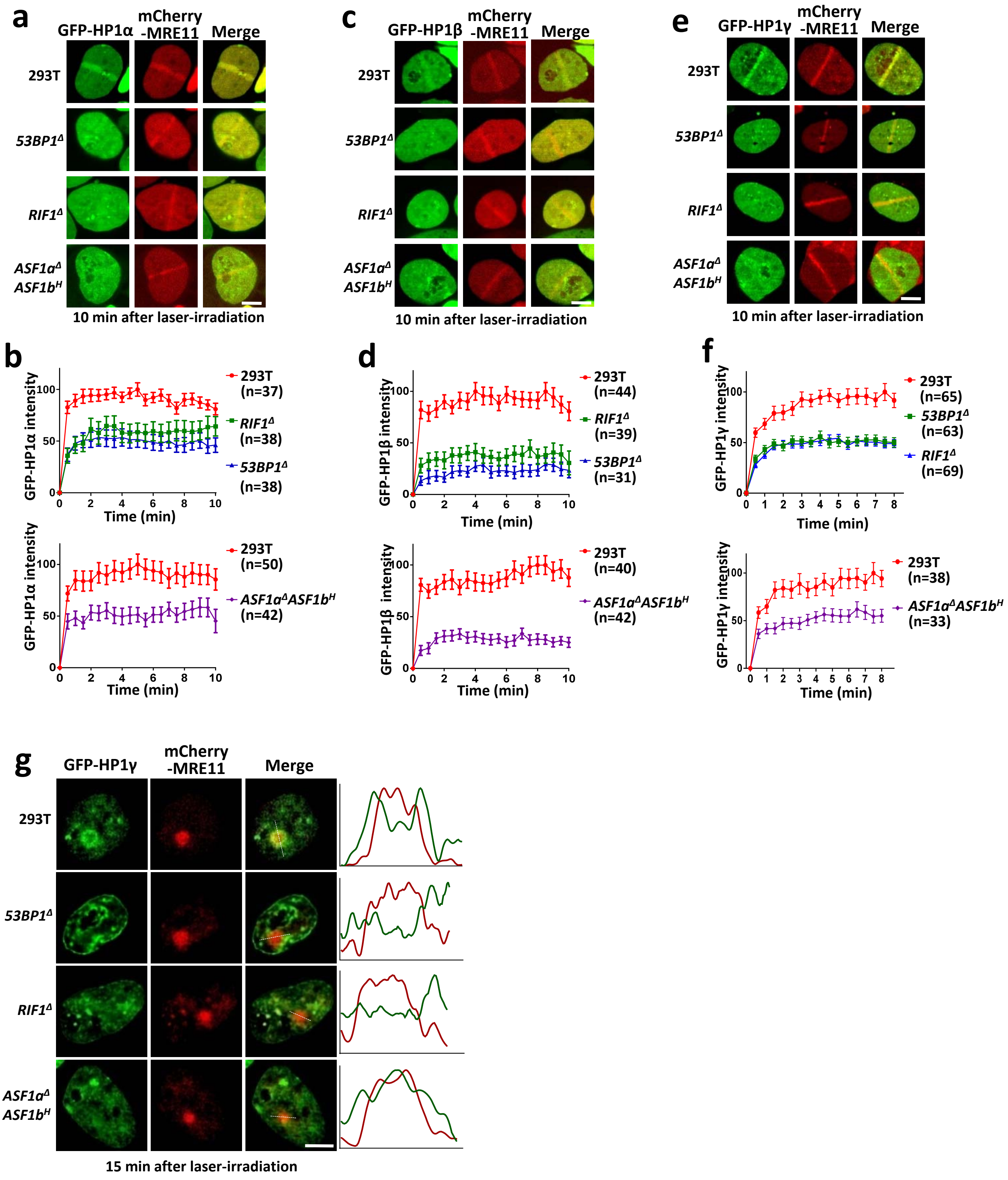

Supplementary Figure 10: HP1 is recruited to DNA damage sites by 53BP1-RIF1-ASF1a/b.

a-f, Recruitment of GFP-HP1α (a), GFP-HP1β (c) and GFP-HP1γ (e) to laser-induced DNA damage sites in wild-type, *53BP1*^{Δ}, *RIF1*^{Δ} or *ASF1a*^{Δ}*ASF1b*^{H} HEK293T cells and quantifications (b, d, f). The mean and SEM values are shown for every time point. **g,** GFP-HP1γ in the HIRDC assay in wild-type, *53BP1*^{Δ}, *RIF1*^{Δ} or *ASF1a*^{Δ}*ASF1b*^{H} HEK293T cells. Scale bar, 5 µm.

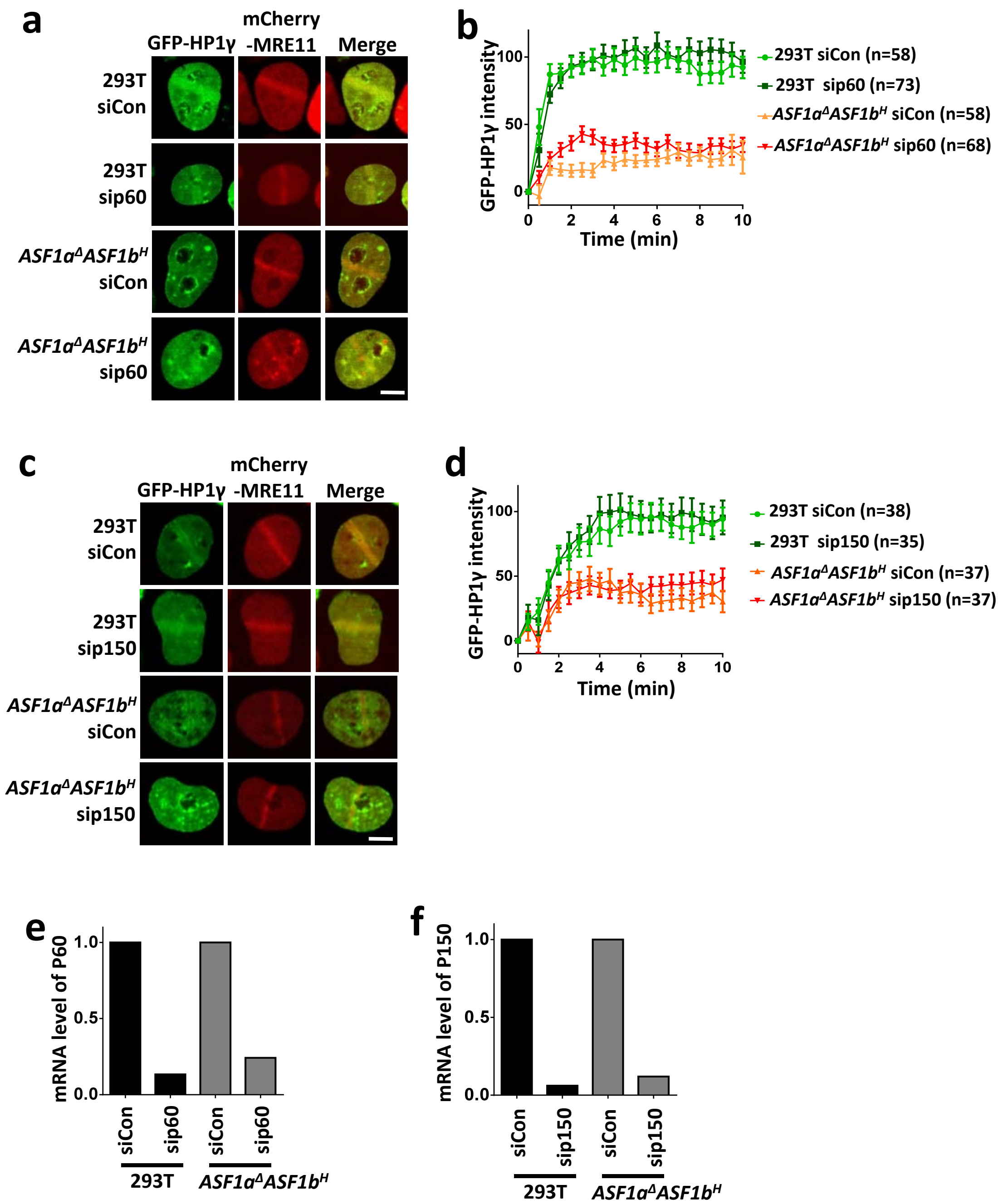

Supplementary Figure 11: CAF-1 is not required for HP1 recruitment to DNA damage sites.

a-d, Recruitment of GFP-HP1γ to laser-induced DNA damage sites in CAF-1 p60 (a) or p150 (c) depleted cells and quantification (b, d). The mean and SEM values are shown for every time point. Scale bar, 5 μm. **e, f,** mRNA levels of CAF-1 p60 (e) and p150 (f) after siRNA knockdown. mRNA levels were measured by RT-PCR. The mean from three technical repeats are shown.

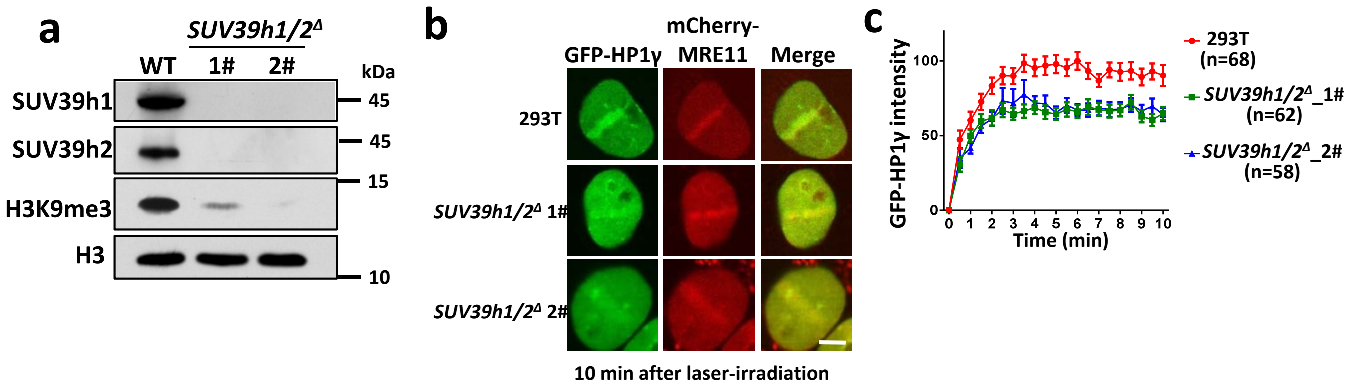

Supplementary Figure 12: SUV39h1/2 promotes the recruitment of HP1 to DNA.

a, Immunoblots showing protein level of SUV39h1 and SUV39h2 in their double knockout HEK293T cells. **b, c,** Image showing the recruitment of GFP-HP1γ to laser-induced DNA damage sites in wild-type and *SUV39h1/2*$^{\Delta}$ HEK293T cells (b) and quantification (c). The mean and SEM values are shown for every time point. Scale bar, 5 µm.

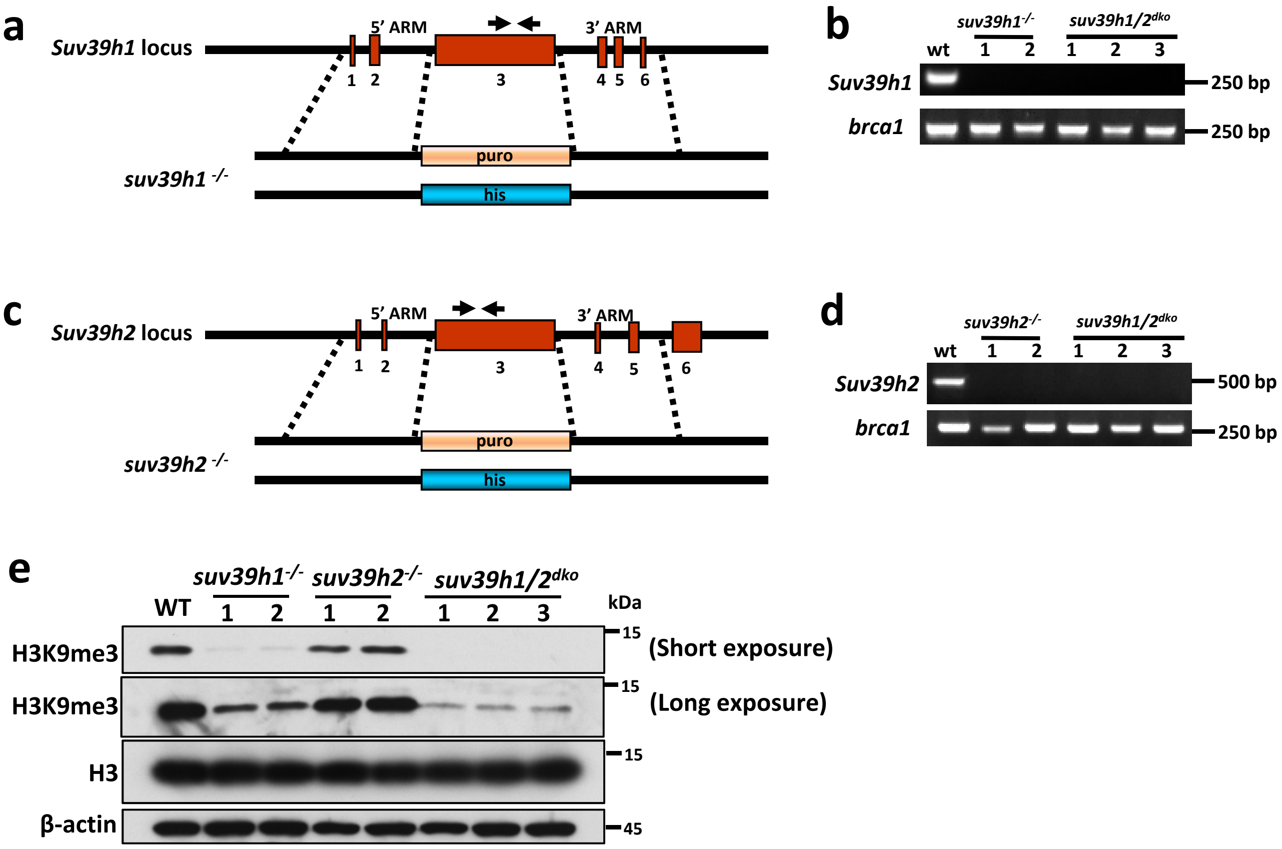

Supplementary Figure 13: Generation of SUV39h1/2 knockout DT40 cells.

a, **c,** Schematic representation of the chicken wild-type and targeted genomic DNA in the *suv39h1* (a) and *suv39h2* (c) genes. Regions containing exons (marked by red) were replaced by histidinol or puromycin resistant genes. The two regions between two pairs of dotted lines were used as the arms of the knock-out constructs. Arrows indicated targeted locations of the primers for genomic PCR. *suv39h1/2^{dko}*, suv39h1 and suv39h2 double knockout cells. **b**, **d,** Genomic PCR analysis to show that *suv39h1* (b; primers: TTCGTCTACATCAACGAGTACAAAGT and AAGATGCAGAGGTCGTAGCGGAT) and *suv39h2* (d; primers: GGAAAGGATGGCCAGAATCTT CAAATACTTGGGAACC and CTGCAAAATGAATTGCATTCATAGATGGGCAAGCC) genes are undetectable in the respective knockout DT40 cells. *Brca1* (primers: CAATTGAGGGCCAGAGTTCTC and GAGAATCATCC ATGGGTTCACAC) was included as a positive control. **e,** Immunoblots showing that H3K9me3 is reduced in the knockout cells.

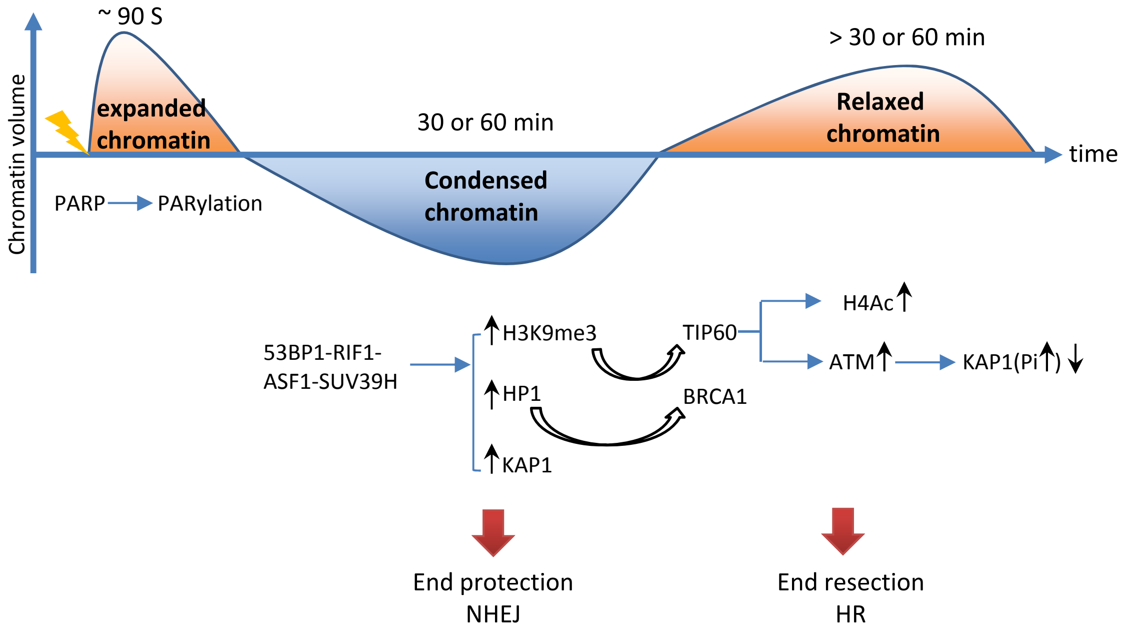

Supplementary Figure 14: A model for chromatin dynamics during DSB repair.

DSB induces a transient chromatin expansion in 90 seconds in a PARylation-dependent manner. Then, the chromatin undergoes a condensation through heterochromatinization mediated by the 53BP1-RIF1-ASF1-SUV39H axis. This condensation protects the broken ends from resection, and thus the DSBs tend to be repair by NHEJ. If rapid repair by NHEJ does not work (in 30 or 60 min), the accumulated heterochromatin marks will recruit TIP60 and BRCA1, which lead to chromatin relaxation and end resection. And then, the DSBs tend to be repaired by HR.
