# Supplementary material for: RIF1-ASF1-mediated high-order chromatin structure safeguards genome integrity
Source: Nat Commun. 2022 Feb 17;13:957. doi: 10.1038/s41467-022-28588-y (PMC8854732; doi:10.1038/s41467-022-28588-y)
Supplement: Supplementary file 3 — Description for Additional Supplementary Files [file 41467_2022_28588_MOESM3_ESM.docx]

**Description of Additional Supplementary Files**

Title: Supplementary Data 1

Description: Mass spectrometry analysis of the mixtures of the indicated immunoprecipitates
