# Supplementary material for: RIF1-ASF1-mediated high-order chromatin structure safeguards genome integrity
Source: Nat Commun. 2022 Feb 17;13:957. doi: 10.1038/s41467-022-28588-y (PMC8854732; doi:10.1038/s41467-022-28588-y)
Supplement: Supplementary file 5 — Reporting Summary [file 41467_2022_28588_MOESM5_ESM.pdf]

nature research

Corresponding author(s): Dongyi Xu

Last updated by author(s): Jan 9, 2022

Reporting Summary

Nature Research wishes to improve the reproducibility of the work that we publish. This form provides structure for consistency and transparency in reporting. For further information on Nature Research policies, see Authors & Referees and the Editorial Policy Checklist .

Statistics

For all statistical analyses, confirm that the following items are present in the figure legend, table legend, main text, or Methods section.

n/a	Confirmed	
☐	☒	The exact sample size (*n*) for each experimental group/condition, given as a discrete number and unit of measurement
☐	☒	A statement on whether measurements were taken from distinct samples or whether the same sample was measured repeatedly
☐	☒	The statistical test(s) used AND whether they are one- or two-sided<br>*Only common tests should be described solely by name; describe more complex techniques in the Methods section.*
☒	☐	A description of all covariates tested
☒	☐	A description of any assumptions or corrections, such as tests of normality and adjustment for multiple comparisons
☐	☒	A full description of the statistical parameters including central tendency (e.g. means) or other basic estimates (e.g. regression coefficient) AND variation (e.g. standard deviation) or associated estimates of uncertainty (e.g. confidence intervals)
☐	☒	For null hypothesis testing, the test statistic (e.g. *F*, *t*, *r*) with confidence intervals, effect sizes, degrees of freedom and *P* value noted<br>*Give P values as exact values whenever suitable.*
☒	☐	For Bayesian analysis, information on the choice of priors and Markov chain Monte Carlo settings
☒	☐	For hierarchical and complex designs, identification of the appropriate level for tests and full reporting of outcomes
☒	☐	Estimates of effect sizes (e.g. Cohen's *d*, Pearson's *r*), indicating how they were calculated

Our web collection on statistics for biologists contains articles on many of the points above.

Software and code

Policy information about availability of computer code

Data collection	ANDOR IQ 3.6.3, Fusion.shell.x64
Data analysis	Prism 6, Microsoft Excel 2013, ImageJ1.49v, Imaris x64 7.4.2, BD FACSuite V1.0.5.3841, FlowJo version X

For manuscripts utilizing custom algorithms or software that are central to the research but not yet described in published literature, software must be made available to editors/reviewers. We strongly encourage code deposition in a community repository (e.g. GitHub). See the Nature Research guidelines for submitting code & software for further information.

Data

Policy information about availability of data

All manuscripts must include a data availability statement. This statement should provide the following information, where applicable:

- Accession codes, unique identifiers, or web links for publicly available datasets
- A list of figures that have associated raw data
- A description of any restrictions on data availability

All relevant data supporting the key findings of this study are available within the article and its Supplementary Information files or from the corresponding author upon reasonable request. Source data are provided with this paper.

Field-specific reporting

Please select the one below that is the best fit for your research. If you are not sure, read the appropriate sections before making your selection.

☒ Life sciences ☐ Behavioural & social sciences ☐ Ecological, evolutionary & environmental sciences

Life sciences study design

All studies must disclose on these points even when the disclosure is negative.

Sample size	No sample size calculation was performed. The number of samples in each experiment was determined based on standards practice in the field. The number of independent experiments are indicated in the legend of each Figure.
Data exclusions	No data was exclused
Replication	All experiments were repeated at least three times and all attempts at replication were successful.
Randomization	Randomization is not relevant because we did not use different experimental groups in our study.
Blinding	Blinding of samples was performed for Fig. 1f-l, 2h-j, 3k-n, 4b-i, 5b-j, 6a-h; and Supplementary Fig.2c and 5a. No other experiments were blinded, since subjective rating of data was not involved.

Reporting for specific materials, systems and methods

We require information from authors about some types of materials, experimental systems and methods used in many studies. Here, indicate whether each material, system or method listed is relevant to your study. If you are not sure if a list item applies to your research, read the appropriate section before selecting a response.

Materials & experimental systems

n/a	Involved in the study	
☐	☒	Antibodies
☐	☒	Eukaryotic cell lines
☒	☐	Palaeontology
☒	☐	Animals and other organisms
☒	☐	Human research participants
☒	☐	Clinical data

Methods

n/a	Involved in the study	
☒	☐	ChIP-seq
☐	☒	Flow cytometry
☒	☐	MRI-based neuroimaging

Antibodies

Antibodies used

Antibody Designation Source or reference Identifiers Additional information Clone name
Asf1a (rabbit polyclonal) Proteintech (China) 22259-1-AP WB:1:2000
IF: 1:200
Asf1b (rabbit polyclonal) Proteintech (China) 22258-1-AP WB:1:2000
Histone3 (rabbit polyclonal) Novus Biologicals (Littleton, USA) NB500-171 WB:1:2000
RIF1 (rabbit polyclonal) homemade WB:1:2000
53BP1 (rabbit polyclonal) Abcam (Cambridge, UK) ab36823 WB:1:2000
53BP1 (rabbit polyclonal) Novus Biologicals (Littleton, USA) NB100-304 IF:1:250
BRCA1 (rabbit polyclonal) Millipore (St. Louis, MO, USA) 07-434 WB: 1:5000 ; IF:1:1000
BRCA1 (mouse monoclonal) Santa Cruz (Dallas, TX, USA) sc-6954 WB:1:100 ; IF:1:40 D-9

RAD51 (rabbit polyclonal) Santa Cruz (Dallas, TX, USA) sc-8349 IF:1:250
RAD51 (rabbit polyclonal) Abcam (Cambridge, UK) ab133534 IF:1:250
γH2AX (mouse monoclonal) Millipore (St. Louis, MO, USA) 05–636 IF:1:5000 JBW301
RPA32 (rabbit polyclonal) Bethyl (Montgomery,TX, USA) A300-244A IF:1:250
Histone H3K9me3 (rabbit polyclonal) Abcam (Cambridge, UK) ab176916 IF:1:1000
Histone H3K9me3 (rabbit polyclonal) Abcam (Cambridge, UK) ab8898 IF:1:250
Brdu (mouse monoclonal) Becton Dickinson 347580 IF:1:50 B44
MRE11(mouse monoclonal) Abcam (Cambridge, UK) ab214 IF:1:250 12D7
SUV39H1 (mouse monoclonal) Millipore (St. Louis, MO, USA) 05-615 WB:1:1000
IF: 1:250 MG44
SUV39H2 (rabbit polyclonal) Abcam (Cambridge, UK) ab190870 WB:1:1000
β-actin (mouse monoclonal) MBL (Japan) M177-3 WB:1:1000 6D1
anti-rabbit IgG Alexa Fluor 594 secondary antibodies Invitrogen A21207 IF:1:250
anti-mouse IgG Alexa Fluor 594 secondary antibodies Invitrogen A21203 IF:1:250
anti-rabbit IgG Alexa Fluor 488 secondary antibodies Jackson Immunoresearch 711-546-152 IF:1:250
anti-mouse IgG Alexa Fluor 488 secondary antibodies Invitrogen A21202 IF:1:250

Validation

Asf1a Proteintech (China) 22259-1-AP validated in WB (1:2000) and IF (1:200) (http://www.ptgcn.com/products/ASF1A-Antibody-22259-1-AP.htm)
Asf1b Proteintech (China) 22258-1-AP validated in WB (1:2000) (http://www.ptgcn.com/products/ASF1B-Antibody-22258-1-AP.htm)
Histone3 Novus Biologicals (Littleton, USA) NB500-171 validated in WB (1:2000) (https://www.novusbio.com/products/histone-h3-antibody_nb500-171)
RIF1 homemade validated in WB (1:2000) (https://pubmed.ncbi.nlm.nih.gov/20711169/)
53BP1 (rabbit polyclonal) Abcam (Cambridge, UK) ab36823 validated in WB (1:2000) (https://www.abcam.com/53bp1-antibody-ab36823.html)
53BP1 (rabbit polyclonal) Novus Biologicals (Littleton, USA) NB100-304 validated in IF (1:250) (https://www.novusbio.com/products/53bp1-antibody_nb100-304)
BRCA1 (rabbit polyclonal) Millipore (St. Louis, MO, USA) 07-434 validated in WB (1:5000) and IF (1:1000) (https://www.sigmaaldrich.cn/CN/zh/search/07-434?focus=products&page=1&perPage=30&sort=relevance&term=07-434&type=product)
BRCA1 (mouse monoclonal) clone D-9 Santa Cruz (Dallas, TX, USA) sc-6954 validated in WB (1:100) and IF (1:40) (https://www.scbt.com/p/brca1-antibody-d-9?requestFrom=search)
RAD51 (rabbit polyclonal) Santa Cruz (Dallas, TX, USA) sc-8349 validated in IF (1:250) (https://www.scbt.com/p/rad51-antibody-h-92?requestFrom=search)
RAD51 (rabbit polyclonal) Abcam (Cambridge, UK) ab133534 validated in IF (1:250) (https://www.abcam.com/rad51-antibody-epr40303-ab133534.html)
γH2AX (mouse monoclonal) clone JBW301 Millipore (St. Louis, MO, USA) 05–636 validated in IF (1:5000) (https://www.sigmaaldrich.cn/CN/zh/substance/antiphosphohistoneh2axser139antibodyclonejbw3011234598765)
RPA32 (rabbit polyclonal) Bethyl (Montgomery,TX, USA) A300-244A validated in IF (1:250) (https://www.biomol.com/products/antibodies/primary-antibodies/general/anti-rpa32-a300-244a-t?number=A300-244A)
Histone H3K9me3 (rabbit polyclonal) Abcam (Cambridge, UK) ab176916 validated in IF (1:1000) (https://www.abcam.com/histone-h3-tri-methyl-k9-antibody-epr16601-chip-grade-ab176916.html)
Histone H3K9me3 (rabbit polyclonal) Abcam (Cambridge, UK) ab8898 validated in IF (1:250) (https://www.abcam.com/histone-h3-tri-methyl-k9-antibody-chip-grade-ab8898.html)
Brdu (mouse monoclonal) clone B44 Becton Dickinson 347580 validated in IF (1:50) (https://www.bdbiosciences.com/en-us/products/reagents/flow-cytometry-reagents/clinical-discovery-research/single-color-antibodies-ruo-gmp/purified-mouse-anti-brdu.347580)
MRE11(mouse monoclonal) clone 12D7 Abcam (Cambridge, UK) ab214 validated in IF (1:250) (https://www.abcam.com/mre11-antibody-12d7-bsa-and-azide-free-ab214.html)
SUV39H1 (mouse monoclonal) clone MG44 Millipore (St. Louis, MO, USA) 05-615 validated in WB (1:1000) and IF (1:250) (https://www.sigmaaldrich.cn/CN/zh/search/05-615?focus=products&page=1&perPage=30&sort=relevance&term=05-615&type=product)
SUV39H2 (rabbit polyclonal) Abcam (Cambridge, UK) ab190870 validated in WB (1:1000) (https://www.abcam.com/kmt1b--suv39h2-antibody-epr18495-ab190870.html)
β-actin (mouse monoclonal) clone 6D1 MBL (Japan) M177-3 validated in WB (1:1000) (https://www.mblbio.com/bio/g/dtl/A/index.html?pcd=M177-3)
anti-rabbit IgG Alexa Fluor 594 secondary antibodies Invitrogen A21207 validated in IF (1:250) (https://www.thermofisher.cn/cn/zh/antibody/product/Donkey-anti-Rabbit-IgG-H-L-Highly-Cross-Adsorbed-Secondary-Antibody-Polyclonal/A-21207)
anti-mouse IgG Alexa Fluor 594 secondary antibodies Invitrogen A21203 validated in IF (1:250) (https://www.thermofisher.cn/cn/zh/antibody/product/Donkey-anti-Mouse-IgG-H-L-Highly-Cross-Adsorbed-Secondary-Antibody-Polyclonal/A-21203)
anti-rabbit IgG Alexa Fluor 488 secondary antibodies Jackson Immunoresearch 711-546-152 validated in IF (1:250) (https://www.jacksonimmuno.com/catalog/products/711-546-152)
anti-mouse IgG Alexa Fluor 488 secondary antibodies Invitrogen A21202 validated in IF (1:250) (https://www.thermofisher.cn/cn/zh/antibody/product/Donkey-anti-Mouse-IgG-H-L-Highly-Cross-Adsorbed-Secondary-Antibody-Polyclonal/A-21202)

Eukaryotic cell lines

Policy information about cell lines

Cell line source(s)	HEK293T, HCT116, DT40 and U2OS cells were obtained from the ATCC. NIH2/4 cells were obtained from Dr. Tom Misteli. U2OS-265 cells were obtained from Roger A. Greenberg.
Authentication	The identity of the cell lines was validated by STR profiling (ATCC) and by analysis of chromosome number in metaphase spreads.
Mycoplasma contamination	All cell lines were subjected to mycoplasma testing twice per month and found to be negative.
Commonly misidentified lines (See ICLAC register)	No cell lines used in this study were found in the database of commonly misidentified cell lines that is maintained by ICLAC and NCBI Biosample

Flow Cytometry

Plots

Confirm that:

- [x] The axis labels state the marker and fluorochrome used (e.g. CD4-FITC).
- [x] The axis scales are clearly visible. Include numbers along axes only for bottom left plot of group (a 'group' is an analysis of identical markers).
- [x] All plots are contour plots with outliers or pseudocolor plots.
- [x] A numerical value for number of cells or percentage (with statistics) is provided.

Methodology

Sample preparation	DT40 cell were cultured in RPMI 1640 medium with 10% fetal bovine serum (FBS; Invirtogen, 2% chicken serum, 10mM HEPES and 1% penicillin-streptomycin mixture at 39.5℃ (5% CO_2). U2OS cells were cultured in DMEM medium supplemented with 10% FBS and 1% penicillin-streptomycin at 37℃ (5% CO_2).<br><br>Cells were labeled with 10 μM BrdU (Sigma; B5002) for 20 min before harvest. After fixation with 70% ethanol, cells were resuspended in 2 ml 4 M HCl at room temperature for 20 min and then neutralized with 10 ml 0.1 M $Na_2B_4O_7$. Then cells were washed twice with 0.1% Triton-100 and 1% BSA in PBS, and were incubated with anti-BrdU antibody (Becton Dickinson; 347580) at room temperature for 30 min. After washing, the cells were incubated with anti-mouse IgG Alexa Fluor 488 secondary antibodies at room temperature for 30 min. After 3 times washing, cells were incubated with 50 μg/ml PI at room temperature for 30min and finally analyzed with BD FACSVerse flow cytometer.
Instrument	BD FACSuite
Software	BD FACSuite V1.0.5.3841; FlowJo version X
Cell population abundance	Sorting was not used in this study.
Gating strategy	After removing doublet, cells were gated SSC-H and FSC-H to obtain the cells. Then, target cell population were analyzed by BrdU and PI staining.

- [x] Tick this box to confirm that a figure exemplifying the gating strategy is provided in the Supplementary Information.
